# Affective language and transdiagnostic symptom burden in clinically referred adolescents: a multicenter Turkish sentence-completion study

**DOI:** 10.3389/fpsyg.2026.1958650

**Published:** 2026-09-21

**Authors:** Zeynep Seda Albayrak, Herdem Aslan Genç, Ezgi Eymen Ekici, Tunahan Tayyip Görgülü, Dilara Demirpençe Seçinti, Gülçin Kahraman, Şafak Eray Çamlı, Hale Yapıcı Eser

**Affiliations:** 1 Department of Child and Adolescent Psychiatry, Koç University Faculty of Medicine, Istanbul, Türkiye; 2 Koç University Research Center for Translational Medicine (KUTTAM), Istanbul, Türkiye; 3 Clinic of Child and Adolescent Psychiatry, Şişli Hamidiye Etfal Training and Research Hospital, Istanbul, Türkiye; 4 Department of Child and Adolescent Psychiatry, Faculty of Medicine, Bursa Uludağ University, Bursa, Türkiye; 5 Department of Psychiatry, Faculty of Medicine, Koç University, Istanbul, Türkiye

**Keywords:** adolescents, affective language, anger, Linguistic Inquiry and Word Count (LIWC), negative emotion, Sentence Completion, transdiagnostic psychopathology, Turkish

## Abstract

Self-report scales dominate adolescent mental-health assessment but depend on symptom recognition and disclosure. Participant-generated language offers a complementary window on affective processes, yet most evidence comes from adult, English-language, community, or social-media data, leaving clinically referred adolescents and structurally different languages such as Turkish understudied. Sentence-completion tasks are clinically familiar but interpreted qualitatively, yielding no quantitative index. In this multicenter cross-sectional study, 445 clinically referred adolescents (aged 10–18 years; 55.1% girls) from three Turkish child and adolescent psychiatry outpatient clinics completed a 24-item sentence-completion task, the Children’s Depression Inventory (CDI), and the Strengths and Difficulties Questionnaire (SDQ). Completions were analyzed with a Turkish Linguistic Inquiry and Word Count (LIWC) dictionary at whole-response and thematic levels. Because anger and sadness words are nested within the broader negative-emotion category, negative-emotion language was separated into anger, sadness, and other negative emotion, so that each coefficient referred lexical items. Partial correlations and hierarchical regressions related these components, positive-emotion language, and first-person singular reference to symptom severity, controlling for age, sex, word count, and study site, with false-discovery-rate correction. Negative-emotion language was positively associated with depressive symptoms, total difficulties, emotional symptoms, and conduct problems (*r* = 0.25 to 0.35), whereas positive-emotion language showed inverse associations. After decomposition, the affective-language block explained 8.1–13.3% of incremental variance, and anger and sadness each contributed beyond general negative tone. The anger coefficient exceeded the sadness coefficient in the conduct-problems model but not in the depressive or emotional-symptoms models. First-person singular reference, which included Turkish verbal morphology and tracked referential context, was unrelated to symptom severity, and no cognitive-process, third-person, or social-content category yielded a detectable association in exploratory screening. The negative-emotion–depression association was of comparable magnitude at all three sites, and the findings persisted after diagnostic adjustment and without the minimum word-count criterion. Affective language in brief Turkish sentence-completion responses therefore tracked dimensional symptom burden, while the absence of a first-person effect under a morphology-informed operationalization underscores the need for cross-linguistically valid measures of self-reference. Automated analysis offers a way of rendering a clinically familiar task quantifiable, as a complementary research measure rather than a standalone diagnostic tool.

## 1 Introduction

Adolescence is a developmental period in which emotional and behavioral symptoms frequently overlap across conventional diagnostic boundaries. Standardized self-report scales remain central to clinical assessment, but they depend on adolescents recognizing and disclosing their difficulties as symptoms and may not capture how they represent themselves, their relationships, and their emotional experiences. Language provides a complementary window into these processes, because word choice can reflect affective salience and attentional focus without requiring adolescents to label them explicitly as symptoms (Pennebaker et al., 2003). Automated text-analysis methods, including Linguistic Inquiry and Word Count (LIWC), quantify psychologically relevant word categories (Boyd et al., 2022) and have increasingly been used to examine linguistic correlates of depression, anxiety, and other forms of distress (Calvo et al., 2017; Eichstaedt et al., 2018). Much of this literature, however, rests on adult, English-speaking, community, or social-media samples, leaving clinically referred adolescents and languages with substantially different grammatical structures comparatively understudied (Dudău and Sava, 2021).

Affective language is among the most consistently examined linguistic correlates of emotional distress. Cognitive models of depression propose that negative schemas influence how individuals attend to, interpret, and describe their experiences, including their representations of the self (Jacobs et al., 2008). Consistent with this account, adolescents with elevated depressive symptoms produce more negative and fewer positive self-descriptions: in a large adolescent sample, greater use of negative-emotion and sadness-related language and reduced positive self-evaluation accompanied depressive symptoms in an open-ended self-description task (Hards et al., 2020, 2023). Clinical interview research has identified both shared and partially distinct linguistic patterns across depression and anxiety (Stade et al., 2023), and adolescents’ everyday smartphone communication carries related information, with positive-emotion and affiliative language linked to more favorable subsequent mood (Funkhouser et al., 2023; Li et al., 2023). Together, these findings suggest that affective language may index symptom severity dimensionally rather than merely distinguish diagnostic groups.

General negative-emotion language, however, aggregates several emotional categories that may carry different clinical meanings. Anger, sadness, anxiety, and other negative emotions are related but not interchangeable, and their relative expression may help distinguish emotional from behavioral difficulties. Early work found that adolescents with externalizing problems used more direct, intense, and outwardly directed emotional language, whereas internalizing problems were associated with more inwardly focused expression (O’Kearney and Dadds, 2005). This is compatible with the cognitive content-specificity hypothesis, in which thoughts concerning loss, failure, threat, and hostility show partially distinct associations with depression, anxiety, and aggression (Beck et al., 1987; Schniering and Rapee, 2004), and with research linking anger proneness and anger rumination more strongly to externalizing symptoms while sadness-related processes track internalizing difficulties (Peled and Moretti, 2007; Wills et al., 2016). Irritability and anger nevertheless remain transdiagnostic in youth (Leibenluft et al., 2024). The relevant question is therefore not whether anger is exclusive to behavioral problems, but whether anger-related language carries information about conduct difficulties beyond the broader tendency to use negative words.

Testing this question requires attention to how dictionary categories are constructed. In LIWC dictionaries, anger and sadness words are subsets of the broader negative-emotion category (Müderrisoğlu, 2012; Boyd et al., 2022). Entering a total negative-emotion score together with its own constituent subcategories creates a part–whole redundancy: coefficients become difficult to interpret and shared variance is distributed arbitrarily across overlapping predictors. A more transparent approach is to decompose the total into mutually exclusive components — anger words, sadness words, and the remaining negative-emotion words — so that each coefficient refers to a distinct set of lexical items and anger and sadness can be compared explicitly within the same model. The present study adopted this decomposition.

Self-referential language represents another frequently studied linguistic correlate of depression. Meta-analytic and multi-laboratory evidence indicates a small but replicable association between depressive symptoms and first-person singular pronoun use, commonly termed “I-talk” (Edwards and Holtzman, 2017; Tackman et al., 2019; Tølbøll, 2019), generally interpreted as heightened self-focus or rumination. However, I-talk appears to be related to broader negative emotionality rather than being specific to depression (Tackman et al., 2019), and its measurement may not be linguistically equivalent across languages: where subjects can be omitted or encoded morphologically, explicit pronouns represent only a fraction of self-reference. Weaker or absent associations have been reported in Chinese and Romanian samples (Lyu et al., 2023; Trifu et al., 2024), and similar concerns have been raised for English–Spanish comparisons (Ramirez-Esparza et al., 2008). Turkish is both agglutinative and subject-dropping: first-person reference can be conveyed through verbal and possessive suffixes without an explicit equivalent of “I,” so a count restricted to overt forms may underestimate self-reference and fail to reproduce associations reported in English.

The elicitation context also shapes the clinical meaning of affective language. Social-media posts and unconstrained narratives provide ecologically rich language but differ considerably across individuals in topic, audience, length, and communicative purpose. Structured sentence-completion tasks offer an alternative by providing common prompts while allowing participants to generate personally meaningful content, and they are clinically familiar: such instruments are used in child and adolescent clinical and educational settings, including in Türkiye, to explore how a young person represents family, self, fears, and the future (Beier, 1960; Koç et al., 2021). Their interpretation, however, is predominantly qualitative and clinician-dependent, and they yield no standardized numeric index comparable across clinicians, centers, or time points. A clinically familiar, low-burden task that is not quantified is therefore a practical target for automated language analysis, which could yield reproducible indices without adding assessment burden.

Quantitative work in this direction remains limited. Self-description paradigms such as the Twenty Statements Test have shown that negatively valenced self-evaluations accompany adolescent depressive symptoms (Orchard et al., 2018; Hards et al., 2023), and profiles derived from the Beier Sentence Completion Test differentiated Turkish adolescents and young adults with internalizing and externalizing disorders, with fewer positive completions characterizing the internalizing group (Koç et al., 2021). These studies nevertheless relied on human coding or categorical group comparison rather than automated analysis. The responses are also brief, and because LIWC variables are percentages of total words, estimates for infrequent categories such as anger and sadness can become unstable in very short texts (Zhao et al., 2016; Vine et al., 2020). Combining responses across prompts provides a more stable participant-level representation, whereas theme-specific analyses may additionally require presence-versus-absence indicators when distributions contain many zeros.

Despite growing research on adolescent language and mental health, several gaps remain. Existing studies have frequently focused on depression alone (Walker et al., 2023), compared narrowly defined groups (Hards et al., 2020), or analyzed English-language social-media and interview data (Eichstaedt et al., 2018). Turkish studies have shown that depressed adolescents disclose symptom-related content online and that Turkish texts can support automated psychiatric classification (Orhan et al., 2014; Gürbüz et al., 2016), but none has combined a standardized sentence-completion task with Turkish LIWC analysis in a large, clinically referred adolescent sample.

The present multicenter study addressed four questions in a transdiagnostic sample of clinically referred adolescents. First, we examined whether negative- and positive-emotion language across combined sentence-completion responses was associated with depressive symptoms, total difficulties, emotional symptoms, and conduct problems, adjusting for age, sex, word count, and study site. Second, using the non-overlapping decomposition described above, we tested whether anger and sadness language contributed information beyond the remaining negative-emotion words, and whether their contributions differed between emotional and conduct outcomes. Third, we examined first-person singular reference, operationalized to include Turkish verbal morphology. Fourth, exploratory analyses investigated whether negative-emotion language was localized within Family, Self, or Negative-emotion-elicitor themes. The study thus brings dimensional clinical measures, a clinically familiar elicitation task, and automated analysis together in a language for which no such evidence yet exists, clarifying both the potential and the linguistic limits of brief affective-language measures.

## 2 Materials and methods

### 2.1 Study design, participants, and procedure

This multicenter cross-sectional study was conducted at the child and adolescent psychiatry outpatient clinics of Koç University Hospital, Şişli Hamidiye Etfal Training and Research Hospital, and Bursa Uludağ University Faculty of Medicine in Türkiye. Participants were recruited between April 2026 and June 2026 using consecutive sampling during routine outpatient visits. All adolescents meeting the eligibility criteria who attended the participating clinics during the recruitment period were invited, and all those for whom the required consent and assent were obtained were enrolled.

The sample comprised 445 adolescents aged 10–18 years (*M* = 14.17, SD = 2.03), including 245 girls (55.1%) and 200 boys (44.9%). Participants were recruited from Şişli Hamidiye Etfal Training and Research Hospital (*n* = 199, 44.7%), Koç University Hospital (*n* = 137, 30.8%), and Bursa Uludağ University Faculty of Medicine (*n* = 109, 24.5%).

Eligibility criteria included being between 10 and 18 years of age, attending one of the participating outpatient clinics, and having sufficient Turkish reading and writing ability to complete the study measures. Adolescents were not enrolled if they had a documented intellectual disability, an acute psychotic or manic episode, or a neurological or medical condition substantially affecting communication, or if the adolescent or their parent or legal guardian declined to participate.

Participants completed the self-report measures and the sentence-completion task during a single outpatient visit. The instruments were administered in paper-and-pencil format under the supervision of the research team. Participants were given standardized instructions explaining how to complete the task, but no assistance regarding the content of their responses was provided. No formal time limit was imposed. Because the sample was transdiagnostic and diagnostic domains were not mutually exclusive, symptom severity was examined dimensionally rather than by assigning participants to exclusive diagnostic groups.

The study was approved by the Koç University Ethics Committee (approval number 2026.154.IRB3.061) and was conducted in accordance with the Declaration of Helsinki. Institutional permissions for data collection were additionally obtained from Şişli Hamidiye Etfal Training and Research Hospital and Bursa Uludağ University Faculty of Medicine. For participants under 18 years of age, written informed consent was obtained from a parent or legal guardian and written assent was obtained from the adolescent. Participants who had reached 18 years of age provided their own written informed consent, in accordance with Turkish law.

### 2.2 Measures

#### 2.2.1 Sentence-completion task

Language samples were collected using a 24-item sentence-completion task. Each item presented a sentence stem that participants completed in their own words; the stems were presented in Turkish, and illustrative examples in English include “When I was little…,” “My mother…,” and “I wish I….” The task was adapted from the short form of the Beier Sentence Completion Test (Form A), a projective sentence-completion instrument widely used in Turkish clinical and educational practice, the Turkish version of which was adapted by the Ankara University Faculty of Languages, History, and Geography (Beier, 1960; Koç et al., 2021). The projective scoring and interpretive system was not applied; the stems were used solely as a standardized elicitation task to obtain comparable, low-burden free-text language samples that were subsequently analyzed with LIWC. The stems addressed self-perception, family and social relationships, emotional experiences, fears, wishes, and future expectations. Because the instrument is a published adaptation, the item wording is not reproduced here; the content domain and thematic classification of each of the 24 items are provided in Supplementary Table S1.

Only the participant-generated completions were included in the linguistic analyses; the standardized sentence stems were excluded. Handwritten responses were transcribed into electronic text by members of the research team at each site. To reduce transcription error, entry and verification were separated: at every site one team member transcribed the responses and a second team member independently checked the electronic text against the original handwritten forms, with discrepancies resolved by returning to the source document. Obvious spelling errors were corrected when the intended word was unambiguous, while the original content, wording, and grammatical structure were otherwise preserved. When a response was illegible, the participant was asked to clarify it immediately after completing the form, and the clarification was noted on the original form. Proper names were retained as written, and unanswered items remained blank. For whole-response analyses, the available responses to all 24 stems were separated by punctuation and concatenated into a single participant-level text.

#### 2.2.2 Children’s Depression Inventory

Depressive symptoms were assessed using the self-report Children’s Depression Inventory (CDI; Kovacs, 1985) and its validated Turkish version (Öy, 1991). The CDI consists of 27 items rated from 0 to 2 and produces a total score ranging from 0 to 54, with higher scores indicating greater depressive symptom severity. Internal consistency in the present sample was high (Cronbach’s α = 0.89; McDonald’s ω = 0.90). CDI total scores were computed using person-mean prorating: a total was calculated for participants who completed at least 22 of the 27 items by multiplying the mean of the completed items by 27, and was treated as missing when more than five items were incomplete.

#### 2.2.3 Strengths and Difficulties Questionnaire

Emotional and behavioral difficulties were assessed using the self-report Strengths and Difficulties Questionnaire (SDQ; Goodman, 1997) and its validated Turkish version (Güvenir et al., 2008). The SDQ contains 25 items divided into five subscales: Emotional Symptoms, Conduct Problems, Hyperactivity/Inattention, Peer Problems, and Prosocial Behavior. The Total Difficulties score is calculated by summing the first four subscales. The SDQ Total Difficulties, Emotional Symptoms, and Conduct Problems scores were included in the present study, with higher scores indicating greater difficulties. Subscale scores were computed using person-mean prorating when at least four of the five items were available, and the Total Difficulties score was calculated only when all four contributing subscales could be scored.

Internal consistency in the present sample was acceptable for Total Difficulties (α = 0.76, ω = 0.76) and Emotional Symptoms (α = 0.70, ω = 0.71), and low for Conduct Problems (α = 0.47, ω = 0.48). Because coefficient alpha is strongly determined by the number of items, it is of limited informativeness for brief, deliberately heterogeneous scales, and the mean inter-item correlation is more appropriate in this situation (Clark and Watson, 1995; McNeish, 2018; Hayes and Coutts, 2020). For the five-item Conduct Problems subscale, the mean inter-item correlation was 0.148, at the lower bound of the range conventionally recommended for broad constructs, and the five items sample distinct and partly low-base-rate behaviors (temper loss, disobedience, fighting, lying, and stealing). Values in this range are typical of the self-report SDQ rather than specific to this sample; a population study of 10,254 adolescents reported subscale alphas of 0.47 to 0.73 (Bøe et al., 2016; see also Goodman, 2001). The implications for interpretation are considered in section “4.5 Strengths and limitations.”

#### 2.2.4 Clinical diagnoses

Clinical diagnoses were established by child and adolescent psychiatrists on the basis of clinical psychiatric interview, in accordance with DSM-5-TR criteria. For analysis, diagnoses were grouped into four non-mutually-exclusive domains: attention-deficit/hyperactivity disorder; other neurodevelopmental disorders, including autism spectrum, specific learning, language, and tic disorders; internalizing disorders, including depressive, anxiety, and obsessive–compulsive disorders; and a residual category comprising trauma-related, disruptive, eating, and remaining disorders. Because these domains were not mutually exclusive, a given participant could be represented in more than one domain. A diagnostic-domain burden score, reflecting the number of positive domains, was computed for descriptive purposes only.

### 2.3 Linguistic analysis

The language samples were analyzed using Linguistic Inquiry and Word Count software (LIWC-22; Boyd et al., 2022) with the Turkish LIWC dictionary developed by Müderrisoğlu (2012); 23,371 entries. LIWC calculated the percentage of words within each text that matched the relevant linguistic categories. The dictionary recognized an average of 90.7% of words in the whole-response texts.

The linguistic variables were specified *a priori* rather than selected from the full LIWC category set, for two reasons. Conceptually, the study questions concerned affective and self-referential language, and the categories with the most consistent prior evidence in adolescent depression and distress are negative-emotion, anger, sadness, and positive-emotion words together with first-person singular reference (Edwards and Holtzman, 2017; Tackman et al., 2019; Hards et al., 2023; Stade et al., 2023). Methodologically, the responses were brief, and percentage-based estimates for low-frequency dictionary categories become unstable as texts shorten (Zhao et al., 2016; Vine et al., 2020); restricting the confirmatory analyses to a small, theoretically motivated set therefore limited both the multiple-comparison burden and the risk of unstable estimates. Cognitive-process categories such as insight, causation, and certainty words were deliberately not included among the primary variables, because they index reasoning and narrative elaboration that short, prompt-constrained completions are unlikely to sample adequately (Pennebaker et al., 2003; Kerz et al., 2023). To document that this restriction did not conceal associations elsewhere in the dictionary, the remaining categories were nevertheless examined in an exploratory screening using the same partial-correlation model, reported in section “3.2 Whole-response affective language and symptom severity.”

#### 2.3.1 Decomposition of negative-emotion language

The primary linguistic variables were positive-emotion, negative-emotion, anger, and sadness word percentages. In the Turkish LIWC dictionary, as in other LIWC dictionaries, anger and sadness words are subsets of the broader negative-emotion category. In the present data, anger and sadness together accounted for a mean of 4.95 percentage points of the mean negative-emotion score of 7.49, and no participant’s anger or sadness percentage exceeded the corresponding negative-emotion percentage.

Total negative-emotion percentage was therefore retained for bivariate analyses, to preserve comparability with the existing literature, but was decomposed into three operationally non-overlapping components for multivariable modeling: anger words, sadness words, and other negative-emotion words (the negative-emotion percentage remaining after anger and sadness were removed). A small number of dictionary entries are classified in more than one subcategory, so the decomposition is not exactly disjoint at the lexical level; the residual overlap was trivial, producing negative values for three participants that were bounded at zero and that never exceeded 0.01 percentage points. Each regression coefficient therefore refers to an essentially distinct set of lexical items rather than to nested categories, and the anger and sadness coefficients can be compared directly within the same model.

#### 2.3.2 First-person singular reference

First-person singular reference was examined as a secondary variable to evaluate the cross-linguistic generalizability of previously reported I-talk associations. In the Turkish LIWC dictionary (Müderrisoğlu, 2012), first-person singular reference is represented by two complementary categories: overt forms (the pronoun-based category comprising ben, bana, beni, benim, kendim, and related forms; 22 entries) and first-person verb inflection (2,647 morphologically marked verb forms). The first-person index used here (TotalI) aggregates both and therefore captures first-person reference conveyed through verbal morphology in addition to overt pronouns. Overt first-person pronouns were near-absent in most responses (*M* = 0.38% of words; zero in 78% of participants), consistent with both the pro-drop character of Turkish and the sentence-completion format, in which the stems themselves establish the referent.

#### 2.3.3 Whole-response and theme-based analyses

For each participant, the responses to the 24 sentence stems were concatenated and analyzed as a single text. Primary whole-response analyses were restricted to participants whose combined responses contained at least 25 words (*n* = 423), to reduce the instability of percentage-based linguistic measures in very short texts; this threshold reflects evidence that dictionary-based category estimates become more stable as text length increases (Zhao et al., 2016; Vine et al., 2020). Because anger and sadness word percentages were positively skewed, square-root-transformed values were used in correlation and regression analyses; untransformed values were retained for descriptive reporting.

Secondary exploratory analyses examined whether negative-emotion language was concentrated within particular thematic contexts. Three conceptually defined themes were examined: Family (responses concerning the participant’s mother, father, siblings, and wishes regarding their parents); Self (responses to self-referential stems, for example those beginning “I wish I…” or “Compared with others, I…”); and Negative-emotion elicitors (responses concerning experiences that made the participant feel sad, afraid, disgusted, or ashamed). The assignment of each item to a theme is given in Supplementary Table S1. For each theme, the relevant responses were concatenated and analyzed separately, and theme-level analyses included participants who contributed at least five words to the relevant theme. Because many Family and Self texts contained no negative-emotion words, negative-emotion language was represented both as a continuous percentage and as a binary indicator reflecting the presence or absence of at least one negative-emotion word; the two indicators were evaluated separately. Sensitivity analyses repeated the binary analyses at minimum theme word-count thresholds of 7, 10, and 12 words.

### 2.4 Statistical analysis

Analyses were conducted using IBM SPSS Statistics, version 28.0. Categorical variables are reported as frequencies and percentages, and continuous variables as means and standard deviations. Item-level missing responses were handled using person-mean prorating rather than case deletion, as described in sections “2.2.2 Children’s Depression Inventory” and “2.2.3 Strengths and Difficulties Questionnaire”. Missing scale scores were not imputed, and analyses used analysis-specific listwise deletion, so the analytic sample size varied across analyses. Sex was entered as a binary variable coded 1 = female and 2 = male, as recorded in the source dataset. For a dichotomous predictor this coding is equivalent to 0/1 dummy coding: the regression coefficient, its standard error, and all model statistics are identical, and only the model intercept differs. The sex coefficients reported in the regression models and in Supplementary Table S2 therefore represent the difference in the outcome for boys relative to girls.

Associations between whole-response linguistic variables and CDI total, SDQ Total Difficulties, SDQ Emotional Symptoms, and SDQ Conduct Problems were examined using partial correlations controlling for age, sex, total word count, and study site, with study site represented by two dummy-coded variables.

Hierarchical linear regressions were conducted for CDI total, SDQ Emotional Symptoms, and SDQ Conduct Problems. SDQ Total Difficulties was examined only at the correlational level, as a global composite index, to avoid redundancy with its constituent subscales. Age, sex, total word count, and study site were entered in Block 1. The four non-overlapping affective-language variables – other negative emotion, positive emotion, square-root-transformed anger, and square-root-transformed sadness – were entered simultaneously in Block 2. Incremental explained variance was evaluated using the change in R^2^, and multicollinearity was assessed using variance inflation factors. Within each model, the anger and sadness coefficients were compared using a Wald test of the linear contrast between them, estimated on the standardized solution so that the comparison concerns the relative strength of the two associations rather than a difference between variables measured on different scales.

Regression assumptions were examined for each model. Residuals were approximately normal for the Emotional Symptoms model and showed mild positive skew for the CDI and Conduct Problems models, consistent with the bounded distributions of these symptom scores; Breusch–Pagan tests indicated minor heteroscedasticity in all three models, and no observation exceeded conventional influence thresholds (all Cook’s distances < 0.05). All regression inference therefore used heteroscedasticity-consistent (HC3) standard errors (Long and Ervin, 2000); ordinary least-squares standard errors are additionally reported in Supplementary Table S2 for completeness. First-person reference (TotalI) was examined separately and was not entered into the affective-language regression models.

Three sets of robustness analyses were conducted. First, the whole-response partial correlations were repeated with the four diagnostic-domain indicators added to the covariates. Second, all whole-response analyses were repeated without the minimum word-count criterion. Third, because the study was multicenter, the principal partial correlations were re-estimated separately within each site.

Theme-level associations were examined using partial correlations controlling for age, sex, the relevant theme word count, and study site. Within each theme, the continuous negative-emotion percentage and the binary presence indicator were examined separately in relation to CDI total, SDQ Emotional Symptoms, and SDQ Conduct Problems.

All tests were two-tailed, with α = 0.05. The Benjamini–Hochberg procedure was used to control the false discovery rate (Benjamini and Hochberg, 1995) within five pre-specified confirmatory analysis families: whole-response partial correlations (20 tests), the affective-language regression coefficients (12 tests), the anger-versus-sadness contrasts (3 tests, one per outcome), continuous theme-level analyses (9 tests), and binary theme-level analyses (9 tests). The three secondary anger-versus-sadness Wald contrasts were treated as a separate family because they address a different hypothesis from the individual coefficients: not whether each linguistic variable is independently associated with an outcome, but whether the anger and sadness associations differ in magnitude. A sixth family was defined for the exploratory dictionary-wide screening described in section “3.2 Whole-response affective language and symptom severity.” This family was not pre-specified, was corrected separately rather than pooled with the confirmatory families, and is reported as exploratory throughout; it was intended to establish that the *a priori* restriction of the primary analyses had not concealed associations elsewhere in the dictionary, not to test hypotheses of equal standing with the confirmatory analyses. Both unadjusted *p*-values and false-discovery-rate-adjusted q-values are reported.

No *a priori* power analysis was conducted, as the sample was assembled through consecutive clinical recruitment rather than to a pre-specified target. A sensitivity analysis was therefore performed to establish the effect sizes the design could detect. With the analytic sample available for the regression models (*n* = 416) and five covariates, a two-tailed test at α = 0.05 had 80% power to detect a partial correlation of 0.138, and the four-predictor affective-language block had 80% power to detect an incremental R^2^ of 0.028. The observed effects exceeded these thresholds substantially, whereas the site-stratified analyses were less well powered (detectable partial correlations of 0.27, 0.24, and 0.20 for the three centers, in ascending order of sample size). These values are reported to assist interpretation of the null findings and of the variability across sites, and are not intended as post hoc power estimates for the observed effects.

## 3 Results

### 3.1 Sample characteristics and dictionary coverage

Diagnostic information was available for 438 participants. The non-mutually-exclusive diagnostic domains were distributed as follows: internalizing disorders, *n* = 225 (51.4%); attention-deficit/hyperactivity disorder, *n* = 198 (45.2%); other diagnoses, *n* = 103 (23.5%); and other neurodevelopmental disorders, *n* = 48 (11.0%). Participants met criteria for an average of 1.31 diagnostic domains (SD = 0.57), reflecting the transdiagnostic and comorbid composition of the sample. Mean clinical scores were 16.54 (SD = 9.35) for CDI total, 15.40 (SD = 6.22) for SDQ Total Difficulties, 4.05 (SD = 2.67) for SDQ Emotional Symptoms, and 2.74 (SD = 1.83) for SDQ Conduct Problems.

The concatenated sentence-completion responses contained an average of 67.1 words (SD = 26.2), and the Turkish LIWC dictionary recognized an average of 90.7% of words. Of the 445 participants, 423 (95.1%) produced at least 25 words and met the eligibility criterion for the primary whole-response analyses. Following analysis-specific listwise deletion, the analytic sample ranged from 411 to 421 across the primary analyses. Descriptive statistics are summarized in Table 1, and descriptive statistics for the linguistic variables are provided in Supplementary Table S6.

**TABLE 1:** Sample characteristics and language-sample descriptives.

Characteristic	Value
Age, M (SD), *n* = 445	14.17 (2.03)
Girls / boys, *n* (%)	245 (55.1) / 200 (44.9)
Study site: Şişli / Koç / Uludağ, *n* (%)	199 (44.7) / 137 (30.8) / 109 (24.5)
Internalizing disorders domain, *n* (%)	225 (51.4)
ADHD domain, *n* (%)	198 (45.2)
Other diagnoses domain, *n* (%)	103 (23.5)
Other neurodevelopmental disorders domain, *n* (%)	48 (11.0)
Number of diagnostic domains, M (SD), *n* = 438	1.31 (0.57)
CDI total, M (SD), *n* = 438	16.54 (9.35)
SDQ Total Difficulties, M (SD), *n* = 442	15.40 (6.22)
SDQ Emotional Symptoms, M (SD), *n* = 443	4.05 (2.67)
SDQ Conduct Problems, M (SD), *n* = 443	2.74 (1.83)
Whole-response word count, M (SD), *n* = 445	67.09 (26.24)
LIWC dictionary coverage, M% (SD), *n* = 442	90.68 (5.60)
Participants with whole-response word count ≥ 25, *n* (%)	423 (95.1)

Ns differ across measures because of missing scale scores; scale scores were not imputed, and analyses used analysis-specific listwise deletion. Diagnostic percentages were calculated among the 438 participants with available diagnostic information. The LIWC dictionary coverage value was calculable for 442 of the 445 participants; the remaining three produced no free-text response (word count = 0). Diagnostic domains were non-mutually exclusive and therefore do not sum to 100%. ADHD, attention-deficit/hyperactivity disorder; CDI, Children’s Depression Inventory; LIWC, Linguistic Inquiry and Word Count; SDQ, Strengths and Difficulties Questionnaire.

### 3.2 Whole-response affective language and symptom severity

Partial correlations controlling for age, sex, whole-response word count, and study site are presented in Table 2. Complete data for all variables in this correlation family were available for 411 participants.

**TABLE 2:** Partial correlations between whole-response linguistic variables and clinical outcomes.

LIWC category	CDI total	SDQ total difficulties	SDQ emotional symptoms	SDQ conduct problems
Negative emotion (total)	0.353***	0.335***	0.289***	0.252***
Positive emotion	–0.224***	–0.208***	–0.193***	–0.105*
Anger, square-root transformed	0.222***	0.220***	0.133*	0.252***
Sadness, square-root transformed	0.229***	0.171***	0.173***	0.064
First-person singular reference (TotalI)	–0.012	0.042	0.013	0.041

*n* = 411. Partial correlations controlled for age, sex, whole-response word count, and study site; study site was represented by two dummy-coded variables. The Benjamini–Hochberg false-discovery-rate correction was applied across this family of 20 tests. Of the 16 associations involving negative emotion, positive emotion, anger, and sadness, all survived correction except sadness–Conduct Problems (*q* = 0.241); among those that survived, the largest adjusted *p*-values were anger–Emotional Symptoms (*q* = 0.010) and positive emotion–Conduct Problems (*q* = 0.045). None of the four associations involving first-person singular reference approached significance (all *q* > 0.45). Negative emotion (total) is reported here for comparability with the existing literature; the non-overlapping decomposition used in the regression models is described in section “2.3.1 Decomposition of negative-emotion language.”

*q < 0.05.

***q < 0.001.

Negative-emotion language was positively associated with all four outcomes: CDI total (*r* = 0.35), SDQ Total Difficulties (*r* = 0.34), SDQ Emotional Symptoms (*r* = 0.29), and SDQ Conduct Problems (*r* = 0.25), all *q* < 0.001. Positive-emotion language was inversely associated with each outcome, with correlations ranging from *r* = –0.11 for Conduct Problems to *r* = –0.22 for CDI total, all *q* < 0.05.

Anger and sadness showed different correlational patterns. Anger language was associated with CDI total (*r* = 0.22), Total Difficulties (*r* = 0.22), Emotional Symptoms (*r* = 0.13), and Conduct Problems (*r* = 0.25), all *q* < 0.05. Sadness language was associated with CDI total (*r* = 0.23), Total Difficulties (*r* = 0.17), and Emotional Symptoms (*r* = 0.17), but not with Conduct Problems (*r* = 0.06, *q* = 0.241). First-person singular language was not associated with any outcome (all | r| ≤ 0.04, all *q* > 0.45).

To confirm that restricting the primary analyses to affective and self-referential categories had not overlooked associations elsewhere in the dictionary, the remaining Turkish LIWC categories were screened with the same partial-correlation model, with low-base-rate categories represented as presence indicators and the screening corrected as a sixth, exploratory false-discovery-rate family, separate from the five confirmatory families defined in section “2.4 Statistical analysis” Cognitive-process and linguistic-style categories (cognitive mechanisms, insight, causation, certainty, discrepancy, tentativeness, and negations), third-person reference, and social and content categories (social words, family, human, achievement, work, friends, health, future focus, anxiety, desire, and death) showed no association with any clinical outcome that survived correction. Associations were close to zero throughout (all | r| < 0.12, minimum *q* = 0.503), the largest being a weak inverse association between death-related words and total difficulties. Five of the 76 tests reached nominal significance before correction, which is within the number expected by chance. The full screening, including the coding of each category and all r and q values, is reported in Supplementary Table S9. Because this screening was exploratory and the texts were brief, it should not be taken as evidence that clinically relevant information is exclusive to affective vocabulary; it indicates only that no additional category yielded a detectable association in these data.

Although the Hyperactivity/Inattention and Peer Problems subscales were not part of the pre-specified analyses, they were examined in the same exploratory manner to document the full pattern across the SDQ (Supplementary Table S10). Peer Problems showed the same profile as the pre-specified outcomes, with positive associations for negative-emotion language (*r* = 0.28), anger (*r* = 0.18), and sadness (*r* = 0.14), and an inverse association for positive-emotion language (*r* = –0.17), all surviving correction. No association with Hyperactivity/Inattention survived correction (all | r| ≤ 0.10), despite 45.2% of the sample falling within the attention-deficit/hyperactivity disorder diagnostic domain.

### 3.3 Non-overlapping affective components in hierarchical regression models

Hierarchical linear regressions were estimated for CDI total, SDQ Emotional Symptoms, and SDQ Conduct Problems. Age, sex, whole-response word count, and study site were entered in Block 1, followed by the four non-overlapping affective-language variables in Block 2. Because the predictors no longer overlapped, collinearity was negligible (maximum variance inflation factor = 1.27).

The affective-language block explained significant incremental variance in all three outcomes (Table 3): CDI total, ΔR^2^ = 0.133, *F*(4, 406) = 18.20; Emotional Symptoms, ΔR^2^ = 0.089, *F*(4, 411) = 12.41; and Conduct Problems, ΔR^2^ = 0.081, *F*(4, 411) = 9.16, all *p* < 0.001. The final models explained 25.91% of the variance in CDI total, 25.90% in Emotional Symptoms, and 8.70% in Conduct Problems; the near-identical values for the two internalizing outcomes reflect coincidence rather than a shared model, as the models differ in sample size, in the variance explained by the covariate block, and in the incremental contribution of the affective-language block.

**TABLE 3:** Hierarchical regression models predicting clinical symptom scores, using non-overlapping affective-language components.

Block 2 predictor / model statistic	CDI total, β (q)	SDQ emotional symptoms, β (q)	SDQ conduct problems, β (q)
Other negative emotion	0.137 (0.003)	0.176 (0.001)	0.102 (0.036)
Anger, square-root transformed	0.193 ( < 0.001)	0.106 (0.019)	0.240 ( < 0.001)
Sadness, square-root transformed	0.216 ( < 0.001)	0.154 (0.002)	0.074 (0.147)
Positive emotion	–0.155 (0.002)	–0.137 (0.006)	–0.077 (0.125)
Anger vs. sadness contrast, p (q)	0.721 (0.721)	0.467 (0.700)	0.011 (0.034)
ΔR^2^ for Block 2	0.133***	0.089***	0.081***
Final model R^2^	0.2591	0.2590	0.0870
Adjusted R^2^	0.243	0.243	0.067
Analytic *n*	416	421	421

Block 1 included age, sex, whole-response word count, and two dummy-coded study-site variables. Block 2 entered the four non-overlapping affective-language components simultaneously; other negative emotion denotes negative-emotion words remaining after anger and sadness words were removed. β values are standardized regression coefficients. Inference used heteroscedasticity-consistent (HC3) standard errors. For the Block 2 coefficients, *q*-values are Benjamini–Hochberg false-discovery-rate-adjusted across the family of 12 coefficients. The anger versus sadness contrast is an HC3 Wald test of the difference between the two standardized coefficients within the same model; the three contrasts were corrected as a separate false-discovery-rate family. All Block 2 F-change tests were *p* < 0.001. Maximum variance inflation factor = 1.27.

***p < 0.001 for ΔR^2^. Final model R^2^ values are reported to four decimal places for the two internalizing outcomes to show that their near-identical magnitude is coincidental.

Other negative-emotion language was independently associated with all three outcomes (β = 0.10 to.18, all *q* ≤ 0.036), and positive-emotion language was inversely associated with CDI total (β = –0.16, *q* = 0.002) and Emotional Symptoms (β = –0.14, *q* = 0.006) but not with Conduct Problems. Once the part–whole overlap was removed, both anger and sadness carried independent information. Anger was associated with all three outcomes (CDI, β = 0.19; Emotional Symptoms, β = 0.11; Conduct Problems, β = 0.24), whereas sadness was associated with CDI total (β = 0.22) and Emotional Symptoms (β = 0.15) but not with Conduct Problems (β = 0.07, *q* = 0.147).

The two standardized coefficients were compared directly within each model. For Conduct Problems, the anger coefficient exceeded the sadness coefficient (contrast *p* = 0.011, *q* = 0.034). For CDI total (*p* = 0.721, *q* = 0.721) and Emotional Symptoms (*p* = 0.467, *q* = 0.700), the two coefficients did not differ. Anger and sadness language therefore contributed comparably to the two internalizing outcomes, whereas anger contributed more than sadness to conduct problems. This outcome-specific pattern should nevertheless be read with one qualification: the three contrasts were estimated in separate models rather than in a single test of the predictor-by-outcome interaction, so the results establish that the anger–sadness difference was detectable only for conduct problems, not that the difference itself differs reliably across outcomes. Full coefficients, including demographic and study-site covariates and both HC3 and ordinary least-squares standard errors, are reported in Supplementary Table S2.

A three-step version of these models, in which anger and sadness were entered after the general affective-tone variables, is reported in Supplementary Table S5. Anger and sadness together added significant variance beyond other negative-emotion and positive-emotion language for all three outcomes (CDI total, ΔR^2^ = 0.074; Emotional Symptoms, ΔR^2^ = 0.031; Conduct Problems, ΔR^2^ = 0.059; all *p* < 0.001).

### 3.4 First-person singular reference

First-person singular reference was unrelated to every clinical outcome (Table 2). To verify that this null result did not simply reflect an insensitive measure, TotalI was compared across thematic contexts that differ in referential focus. TotalI was substantially higher in self-referential responses (*M* = 18.69%, SD = 12.98) than in responses concerning family members (*M* = 14.34%, SD = 14.12), t(400) = 4.46, *p* < 0.001, dz = 0.22. The index therefore tracked the referential content of the text while showing no association with symptom severity.

### 3.5 Robustness analyses

To examine whether the whole-response associations were independent of recorded diagnoses, the partial correlations were repeated with the four diagnostic-domain indicators added to the covariates. Complete data were available for 409 to 414 participants. The association between negative-emotion language and CDI total decreased from *r* = 0.35 to *r* = 0.30, and the association with Emotional Symptoms decreased from *r* = 0.28 to *r* = 0.22, whereas the association with Conduct Problems was unchanged (*r* = 0.25). The anger–Conduct Problems association was likewise unchanged (*r* = 0.24 to *r* = 0.26). All of these associations remained significant (all *p* < 0.001). To confirm that these changes reflected the diagnostic covariates rather than the accompanying reduction in sample size, the unadjusted coefficients were re-estimated in the diagnosis-complete analytic sample. They were essentially identical to the full-sample estimates (for negative-emotion language and CDI total, *r* = 0.346 in the full sample and *r* = 0.346 in the diagnosis-complete subsample), indicating that the attenuation observed after adjustment is attributable to the diagnostic covariates rather than to sample composition. The corresponding diagnosis-adjusted partial correlations are presented in Supplementary Table S7.

Because the study was multicenter, the principal association was also estimated within each site. The partial correlation between negative-emotion language and CDI total was similar at all three centers: *r* = 0.31 (*n* = 134, *p* < 0.001) at Koç University Hospital, *r* = 0.35 (*n* = 96, *p* < 0.001) at Bursa Uludağ University, and *r* = 0.36 (*n* = 186, *p* < 0.001) at Şişli Hamidiye Etfal Training and Research Hospital. The anger–Conduct Problems association was significant at two of the three sites (*r* = 0.35, *p* < 0.001; *r* = 0.24, *p* = 0.006) and non-significant at the third (*r* = 0.06, *p* = 0.562), which had the smallest subsample. The complete site-stratified partial correlations are presented in Supplementary Table S8.

The analyses were also repeated without applying the minimum word-count criterion (*n* = 430 for the partial correlations, 435 for the CDI regression, and 440 for the SDQ regressions). The overall pattern was unchanged: the affective-language block explained 13.1% of the variance in CDI total, 8.5% in Emotional Symptoms, and 7.6% in Conduct Problems (all *p* < 0.001); anger remained associated with all three outcomes and sadness with CDI total and Emotional Symptoms but not Conduct Problems; and the anger versus sadness contrast for Conduct Problems remained significant (*p* = 0.003). The corresponding models are reported in Supplementary Tables S3, S4.

### 3.6 Theme-based analyses

Secondary exploratory analyses examined negative-emotion language within the Family, Self, and Negative-emotion-elicitor themes, controlling for age, sex, theme-specific word count, and study site.

When negative-emotion language was represented as a continuous percentage, none of the associations survived FDR correction; the lowest adjusted value was *q* = 0.190 (Table 4). Because the theme-level texts were short and frequently contained no negative-emotion words, the analyses were repeated using a binary indicator reflecting the presence or absence of at least one negative-emotion word. One association survived correction: within the Self theme, the presence of at least one negative-emotion word was associated with higher Emotional Symptoms (*r* = 0.16, *q* = 0.032). The unadjusted mean Emotional Symptoms score was 4.39 (SD = 2.81) among adolescents who used at least one negative-emotion word in their self-referential responses (*n* = 169), compared with 3.69 (SD = 2.54) among those who did not (*n* = 190). No binary indicator within the Family or Negative-emotion-elicitor themes was associated with any outcome after correction.

**TABLE 4:** Theme-level negative-emotion language and clinical outcomes.

Theme	Negative-emotion indicator	Analytic *n*	CDI total, r	SDQ emotional symptoms, r	SDQ conduct problems, r
Family	Continuous percentage	400	–0.065	–0.045	–0.115
Self	Continuous percentage	353	0.044	0.085	0.029
Negative-emotion elicitors	Continuous percentage	401	–0.006	–0.032	–0.011
Family	Presence of any negative-emotion word	400	–0.034	–0.021	–0.043
Self	Presence of any negative-emotion word	353	0.072	0.155*	0.037
Negative-emotion elicitors	Presence of any negative-emotion word	401	–0.002	0.008	0.002

Partial correlations controlled for age, sex, theme-specific word count, and study site. Continuous-percentage and binary-indicator analyses were treated as separate false-discovery-rate families of nine tests each. No continuous association survived correction; the lowest adjusted value was *q* = 0.190 for family negative-emotion percentage–Conduct Problems. For the binary analyses, only the association between the presence of negative-emotion language in the Self theme and Emotional Symptoms survived correction (*q* = 0.032). Analytic n reflects listwise deletion across the variables entered in each correlation family; single-outcome models use pairwise deletion and therefore have slightly larger analytic samples (e.g., *n* = 359 for the Self-theme Emotional Symptoms model).

*q < 0.05.

The Self-theme association remained significant after additional adjustment for the four diagnostic domains (*r* = 0.13, *p* = 0.012) and across increasingly restrictive minimum word-count thresholds: *r* = 0.16 (*p* = 0.003, *n* = 359) at ≥ 5 words, *r* = 0.15 (*p* = 0.011, *n* = 276) at ≥ 7 words, and *r* = 0.20 (*p* = 0.016, *n* = 139) at ≥ 10 words. At ≥ 12 words the effect remained in the same direction and of comparable magnitude (*r* = 0.16, *n* = 80) but was no longer statistically significant.

## 4 Discussion

This multicenter study examined whether affective language in sentence-completion responses was associated with symptom severity in a large, transdiagnostic sample of clinically referred adolescents. Four findings emerged. First, greater use of negative-emotion language was consistently associated with depressive symptoms, total difficulties, emotional symptoms, and conduct problems, whereas positive-emotion language showed inverse associations. Second, when negative-emotion language was decomposed into non-overlapping components, anger and sadness each carried information beyond the remaining negative-emotion words, and the anger coefficient exceeded the sadness coefficient for conduct problems but not for depressive or emotional symptoms. Third, dictionary-detected first-person singular reference was unrelated to symptom severity, even though the index was demonstrably sensitive to referential context. Fourth, theme-level analyses provided only limited evidence for contextual localization. The association between negative-emotion language and depressive symptoms was of comparable magnitude at all three sites (*r* = 0.31, 0.35, and 0.36), persisted after adjustment for overlapping diagnostic domains, and was not determined by the minimum word-count criterion. Site-stratified estimates for the remaining associations were more variable, and the anger–conduct association in particular was small at one center; these secondary estimates are based on subsamples of 109 to 199 participants and are correspondingly imprecise.

### 4.1 Affective language as a dimensional correlate of symptom burden

The most consistent finding was the association between whole-response negative-emotion language and all four clinical outcomes. This accords with cognitive accounts proposing that emotional difficulties influence the accessibility and salience of negative representations, which may in turn shape how individuals describe themselves and their experiences (Jacobs et al., 2008; Gotlib and Joormann, 2010). In adolescent self-description tasks, elevated depressive symptoms have similarly been associated with more negative-emotion and sadness-related language and fewer positively valenced self-evaluations (Orchard et al., 2018; Hards et al., 2023), and clinical interview research has identified increased negative-emotion language as a feature shared across depression and anxiety (Stade et al., 2023). The present findings extend this literature by demonstrating dimensional associations within a heterogeneous clinical sample rather than by comparing depressed and non-depressed groups.

The breadth of the associations is noteworthy. Negative-emotion language was related not only to CDI scores and SDQ Emotional Symptoms but also to Total Difficulties and Conduct Problems, suggesting that it may index a broad burden of distress or dysregulation rather than a feature specific to depression, consistent with evidence that linguistic markers commonly associated with depression partly reflect general negative emotionality (Tackman et al., 2019). Adjustment for the recorded diagnostic domains attenuated some associations but did not eliminate them, and the negative-emotion–depression association was of comparable magnitude at all three centers. The results therefore cannot be explained simply by adolescents with depressive or anxiety diagnoses producing more negative words, nor by the characteristics of a single site.

This diagnosis-adjusted finding should nevertheless be interpreted cautiously. The diagnostic domains were broad, non-mutually exclusive, and did not capture variation in current symptom severity, episode status, treatment exposure, or functional impairment, so persistence after adjustment does not establish that the language measures are independent of psychopathology. It supports a more limited conclusion: affective language was associated with dimensional symptom burden over and above the information represented by the available categorical diagnostic indicators.

The incremental variance explained by the affective-language block ranged from 8.1 to 13.3%. At the group level, this represents a meaningful contribution for a brief, low-burden language task, and is of the order reported for other lexicon-based mental-health indicators (Calvo et al., 2017; Eichstaedt et al., 2018). These effect sizes are nevertheless not sufficient to support individual-level screening or diagnostic use, a distinction that is particularly important for Conduct Problems, for which the final model explained less than 10% of the total variance. The language variables are therefore better understood as complementary correlates that may add information to established assessment methods than as standalone indicators of clinical status.

Positive-emotion language showed an inverse pattern, remaining independently associated with CDI total and Emotional Symptoms after the other affective components were entered. This is compatible with adolescent studies linking more positive language to more favorable mood states and lower internalizing symptoms (Li et al., 2023; McNeilly et al., 2023) and with evidence that depressed adolescents produce fewer positively valenced self-descriptions (Orchard et al., 2018; Hards et al., 2023). Positive-emotion word frequency should not, however, be treated as a direct measure of positive affect or anhedonia: reduced positive language could equally reflect a generally negative response style or differences in the topics selected. These inverse associations are accordingly better described as linguistic correlates of lower symptom burden than as evidence that positive-emotion language is itself protective.

An equally informative feature of the results is where signal was absent. In the exploratory screening, cognitive-process categories (cognitive mechanisms, insight, causation, and certainty), third-person reference, and social and content categories showed no association with any outcome, so the detectable information in these brief texts was concentrated in affective vocabulary. Two explanations are plausible and not mutually exclusive. Cognitive-process markers describe how a speaker reasons about and elaborates an experience, features that require sustained narrative and are unlikely to be sampled by single-clause completions averaging 67 words in total (Pennebaker et al., 2003; Kerz et al., 2023), whereas emotion words can be conveyed in a few words and were correspondingly detectable. The prompt structure may compound this, because stems that name a topic elicit content rather than reasoning about it. This pattern is therefore best read as a property of the elicitation format rather than as evidence that cognitive-process language is uninformative in adolescent psychopathology, and it delimits what a brief sentence-completion sample can be expected to measure.

A comparable boundary emerged across clinical domains. Affective language was associated with peer difficulties as strongly as with the pre-specified outcomes, but showed no association with hyperactivity and inattention, even though the ADHD domain was the second most common in this sample. Emotion words therefore do not appear to index general clinical severity, which would have produced associations across all domains. The pattern is more consistent with a specific link to affective and interpersonal difficulties, and the absence of an association with hyperactivity constitutes a form of discriminant evidence rather than a failure of the measure.

### 4.2 Anger and sadness: a partial, outcome-specific differentiation

The clearest differentiated finding concerned anger and sadness. At the bivariate level, anger words correlated with all four clinical outcomes, indicating that anger-related expression was not exclusive to behavioral difficulties. This is consistent with contemporary accounts of irritability and anger as transdiagnostic phenomena occurring across depressive, anxiety, disruptive, and neurodevelopmental conditions in youth (Stringaris et al., 2009; Klein et al., 2021; Leibenluft et al., 2024; Chin et al., 2025). Sadness words, by contrast, correlated with depressive, total, and emotional difficulties but not with conduct problems.

The multivariable models clarified this pattern. Because anger and sadness words are lexical subsets of the negative-emotion category (Boyd et al., 2022), entering a total negative-emotion score alongside its own subcategories would confound part with whole. When the total was instead decomposed into non-overlapping components, both anger and sadness contributed information beyond the remaining negative-emotion words for depressive and emotional symptoms, while only anger did so for conduct problems. Directly comparing the two standardized coefficients confirmed this asymmetry: the anger coefficient exceeded the sadness coefficient for conduct problems, whereas the two were indistinguishable for depressive and emotional symptoms. Specific emotion words therefore add to what general negative tone conveys, and anger carries information about conduct difficulties that sadness does not. Because each contrast was estimated within its own model, however, the results show where the anger–sadness difference was detectable rather than establishing that the difference itself varies reliably across outcomes; a direct test of the predictor-by-outcome interaction would be required for that stronger claim.

This pattern is consistent with earlier work showing that adolescents with externalizing disorders use more direct, intense, and outwardly oriented emotional language, whereas those with internalizing disorders show comparatively inwardly focused and elaborated emotional responses (O’Kearney and Dadds, 2005). It is also compatible with the cognitive content-specificity model, in which hostility- and revenge-related cognitions show stronger associations with aggression while loss and failure content are more closely linked to depression (Beck et al., 1987; Schniering and Rapee, 2004), and with research connecting anger proneness and anger rumination more closely with externalizing symptoms while acknowledging that anger-related processes also accompany internalizing difficulties (Peled and Moretti, 2007; McLaughlin et al., 2011; Wills et al., 2016).

The study does not demonstrate that anger words are a specific marker of conduct disorder or aggressive behavior. The finding is narrower: among adolescents with comparable levels of other negative-emotion language, relatively greater anger-related content was associated with higher conduct-problem scores, and this advantage over sadness was specific to that outcome. Existing linguistic research provides only partial parallels. Mobile-sensed language studies indicate that anger-related categories show both overlap with and differentiation from broader negative-emotion, anxiety, and sadness language (Carlier et al., 2022), and emotion-word profiles in parent–child communication have been linked to callous-unemotional and conduct-related characteristics, although in younger samples (Waller et al., 2024). Much of the broader evidence concerns irritability or aggression rather than spontaneous anger-word use.

The sadness findings warrant a parallel caution. Sadness language was associated with depressive and emotional symptoms independently of other negative-emotion words, consistent with research linking sadness-related words, negative self-evaluations, and depressive symptoms in adolescents (Rude et al., 2004; Symons, 2017; Hards et al., 2023) and with interview findings identifying sadness language as relatively characteristic of depression (Stade et al., 2023). It did not, however, distinguish depressive from broader emotional symptoms. Depressive and emotional difficulties may be represented through a wide range of negative evaluations involving worthlessness, failure, fear, guilt, loneliness, or hopelessness, many of which fall outside the narrower sadness category. Measurement characteristics may also contribute, since sadness words were infrequent and positively skewed, and in short texts a single word substantially changes a percentage-based score (Zhao et al., 2016; Vine et al., 2020).

### 4.3 First-person singular reference and the limits of cross-linguistic I-talk measures

Contrary to much of the English-language literature, dictionary-detected first-person singular reference was unrelated to depressive, emotional, conduct, or total-difficulty scores. Meta-analytic and multi-study evidence has identified a small association between first-person singular pronoun use and depression, generally interpreted as heightened self-focus or rumination (Edwards and Holtzman, 2017; Tackman et al., 2019; Tølbøll, 2019), but these effects are modest and context-dependent (Pennebaker et al., 2003), and Tackman et al. (2019) found the association substantially reduced once negative emotionality was considered.

Cross-linguistic equivalence is central to interpreting the present null result (Dudău and Sava, 2021). Turkish is an agglutinative, subject-dropping language in which grammatical person can be encoded through verbal and possessive morphology without an overt subject pronoun. Null subjects are common once the discourse referent has been established, and person agreement on the verb preserves the identity of the speaker without an independent form equivalent to “I” (Ögel-Balaban and Aksu-Koç, 2020; Uygun, 2022). Overt first-person subjects may additionally serve discourse-pragmatic functions such as emphasis or contrast rather than functioning as neutral indicators of self-reference (Çeltek, 2020). Their frequency therefore does not necessarily carry the same psychological interpretation in Turkish as in English, and a measure restricted to overt forms would index discourse-pragmatic marking as much as self-focus.

Two features of the present operationalization constrain how the null result should be read. On the one hand, the index was not restricted to overt pronouns: TotalI aggregates the 22-entry overt pronoun category with 2,647 morphologically marked first-person verb forms (Müderrisoğlu, 2012), and because overt pronouns were near-absent (*M* = 0.38% of words; zero in 78% of participants), it was driven almost entirely by verb morphology. The null association therefore cannot be attributed to counting overt pronouns while ignoring morphologically encoded self-reference, and the index behaved as expected with respect to referential content, being markedly higher in self-referential than in family-related responses. On the other hand, a rule-based dictionary matching inflected verb forms against a fixed entry list cannot guarantee exhaustive morphological coverage of an agglutinative language, and residual measurement error remains plausible. The appropriate conclusion is therefore narrow: first-person singular reference, as operationalized by TotalI, was not associated with symptom severity in this sample, which does not demonstrate that self-focus more broadly is unrelated to adolescent distress in Turkish.

Several further factors may have contributed. The sentence-completion format itself may suppress overt first-person pronouns, because several stems already establish a first-person referent (e.g., “I wish I…,” “When I was little…”). The sample consisted entirely of clinically referred adolescents, which may have restricted variation in distress-related self-focus (Ingram, 1990), and the expected I-talk effect is small even in meta-analytic English-language findings. Cross-linguistic studies in other pro-drop languages have likewise produced weaker or absent associations (Ramirez-Esparza et al., 2008; Lyu et al., 2023; Trifu et al., 2024). Future Turkish-language work should compare TotalI with human-coded self-reference and with morphology-aware computational measures that identify person marking on verbs and nouns.

### 4.4 Where negativity appears: theme-level findings

The theme analyses provided less consistent evidence than the whole-response analyses. Continuous negative-emotion percentages within the Family, Self, and Negative-emotion-elicitor themes were not associated with clinical outcomes after correction, suggesting that the overall association between affective language and symptom burden was not readily attributable to one narrow thematic context and may be more informative when aggregated across domains of experience. The absence of a continuous association in the Negative-emotion-elicitor theme may partly reflect the structure of the prompts, which constrain the semantic content of the response and may elicit at least some negative language across the symptom distribution. Family responses, in turn, may mix positive, negative, factual, and ambivalent content that a single negative-emotion percentage represents poorly (Vine et al., 2020).

One exploratory finding did survive correction: adolescents who used at least one negative-emotion word in the Self theme reported higher SDQ Emotional Symptoms. The result remained significant after diagnostic adjustment and was similar across several minimum word-count thresholds. This is broadly consistent with cognitive models of negative self-schema and with research showing that adolescents with elevated depressive symptoms produce more negative self-descriptions (Orchard et al., 2018; Hards et al., 2023), with Turkish sentence-completion research finding that reduced positive responding differentiates internalizing from externalizing groups (Koç et al., 2021), and with studies linking self-focused or negatively valenced language to poorer mood (Symons, 2017; Funkhouser et al., 2023; McNeilly et al., 2023). Its specificity to SDQ Emotional Symptoms rather than CDI total may indicate that self-referential negativity relates to a broader internalizing domain, but the association was small, exploratory, and not reproduced across outcomes.

The contrast between the binary and continuous findings is methodologically informative. Family and Self texts were short and frequently contained no negative-emotion words, and under these conditions a percentage score is highly sensitive to its denominator: one word in a five-word response yields a very different percentage from one word in a 15-word response, although both participants expressed some negative content. Short texts and low-base-rate categories are known to produce less stable lexicon-based estimates (Zhao et al., 2016; Calvo et al., 2017; Vine et al., 2020), and generic word-count lexicons may be less informative than context-sensitive features when texts are brief or linguistically heterogeneous (Kerz et al., 2023). The present study nevertheless does not establish that binary presence is generally superior: it avoids unstable distinctions among very small percentages but discards information about frequency and intensity. Comparison with count, proportion, hurdle, or zero-inflated models in longer responses would be valuable.

### 4.5 Strengths and limitations

Several strengths increase confidence in the overall pattern. The sample was relatively large, clinically referred, transdiagnostic, and recruited from three centers, at each of which the negative-emotion–depression association was of comparable magnitude, and the study included depressive, broad emotional, behavioral, and total-difficulty outcomes rather than a single diagnosis. Standardized stems reduced variation in elicitation context, and analyses excluded the stems themselves. Decomposing negative-emotion language into non-overlapping components removed the part–whole redundancy inherent in nested dictionary categories and permitted a direct comparison of anger and sadness. False-discovery-rate correction was applied to all analysis families (Benjamini and Hochberg, 1995), and inference used heteroscedasticity-consistent standard errors (Long and Ervin, 2000).

The findings must nevertheless be considered in light of several limitations. First, the cross-sectional design precludes conclusions about temporal direction: greater symptoms may shape affective language, but recurring language patterns may also reflect broader cognitive or interpersonal processes associated with symptom maintenance. Second, the symptom outcomes were based on adolescent self-report and were collected in the same assessment context as the language task, so shared response tendencies or current mood may have contributed to the observed associations. Third, participants were enrolled consecutively, but the numbers approached, declining, and excluded were not recorded prospectively; a formal participant flow diagram therefore cannot be provided, and the possibility of unmeasured selection cannot be excluded.

Fourth, the diagnostic domains were broad and overlapping. Although this reflected the clinical reality of the sample and supported dimensional analysis, it limited disorder-specific interpretation. Fifth, CDI, SDQ Total Difficulties, and SDQ Emotional Symptoms were themselves correlated and should not be viewed as independent replications of the same language association. Sixth, LIWC is a context-insensitive dictionary method: it quantifies predefined categories but does not determine who experienced the emotion, whether the statement was hypothetical, or whether a word was negated. Seventh, the language samples were brief (*M* = 67.1 words), and theme-level texts were shorter still and zero-inflated, reducing the stability of continuous LIWC percentages; the binary Self finding was robust across several word-count thresholds but remains exploratory. Text length constrains detection in two distinct ways. The first is statistical: because LIWC variables are percentages of total words, a category occurring at a low base rate is estimated from very few tokens, so its score is dominated by whether a given word happened to appear rather than by how characteristic that category is of the participant. Such measurement error attenuates observed associations, and the shorter the text, the greater the attenuation. The second is linguistic: some categories require discourse that a single-clause completion cannot contain. Cognitive-process markers such as insight, causation, and certainty words index how a speaker reasons about and elaborates an experience, and reasoning of this kind unfolds across several clauses. A response of two or three words can readily express an emotion but cannot easily express a causal explanation of it. The null results for cognitive-process, third-person, and social-content categories should therefore be interpreted as specific to this elicitation format. Given the sensitivity analysis reported in section “2.4 Statistical analysis,” associations of the magnitude observed for the affective categories would have been detected had they been present; associations substantially weaker than that threshold would not. These results accordingly indicate that the categories carried no detectable signal in brief prompt-elicited responses, not that they are uninformative in longer or more open-ended language samples.

Eighth, the internal consistency of the five-item SDQ Conduct Problems subscale was low (α = 0.47, ω = 0.48). Measurement error in an outcome attenuates observed associations and reduces power rather than inflating the false-positive rate (Schmidt and Hunter, 1996), so the conduct-related coefficients reported here are more likely to underestimate than to overestimate the underlying associations. At the same time, the Conduct Problems score is a noisy indicator of the construct, and the modest variance explained by that model (8.7%) should be read accordingly.

A further consideration is the source of the behavioral ratings. Conduct problems were assessed by adolescent self-report alone, and adolescents tend to report externalizing behavior less readily than parents or teachers do. Under-reporting of this kind restricts variance and introduces informant-specific error, and may therefore have contributed both to the low internal consistency of the subscale and to the modest variance explained by that model. This limitation bears directly on the anger–sadness comparison, which is the most distinctive result reported here. That comparison was significant only in the Conduct Problems model, and Conduct Problems is the least reliably measured of the three outcomes. Low outcome reliability does not generate a spurious difference between two predictors, since both the anger and the sadness coefficients are attenuated by the same outcome error; the concern is rather one of precision. The contrast was estimated with a wider effective confidence interval than the corresponding contrasts for the two internalizing outcomes, and it was the only one of the three to depend on a subscale with unacceptable internal consistency. The direction of the effect is consistent with theory and with the site-stratified and word-count sensitivity analyses, but its magnitude should be treated as imprecisely estimated.

Finally, the sample consisted of adolescents referred to psychiatric outpatient clinics in Türkiye. Findings may not generalize to community samples, younger children, other Turkish-speaking populations, or different elicitation contexts. No independent validation sample or out-of-sample predictive analysis was available.

### 4.6 Implications and future directions

The study supports the feasibility of combining structured sentence-completion tasks with automated language analysis in adolescent clinical settings. Sentence-completion methods occupy a useful middle ground between tightly structured symptom questionnaires and unconstrained social-media or narrative data: common prompts reduce topic-related variability while still allowing participants to generate personally meaningful responses. Their practical appeal is that they are clinically familiar and low in burden, yet are ordinarily interpreted qualitatively and yield no score that can be compared across clinicians or centers. Automated analysis offers a route to deriving reproducible indices from such material, and the associations reported here indicate that these indices carry information about dimensional symptom burden, particularly in settings where sentence-completion tasks are already in use. Previous work using the Twenty Statements Test and related self-description paradigms has shown that open-ended completions can capture clinically relevant differences in self-evaluation (Orchard et al., 2018; Hards et al., 2023), and Turkish work with the Beier Sentence Completion Test has demonstrated associations with internalizing and externalizing profiles (Koç et al., 2021).

The mean dictionary coverage of approximately 91% indicates that Turkish LIWC could process most of the language generated in this task. High coverage should not, however, be equated with construct validity: a word may be recognized without its contextual meaning being correctly represented. LIWC does not fully model negation, irony, idiom, semantic scope, or the target of an emotion, so an anger word directed toward oneself, a parent, or an imagined event receives the same count despite potentially different clinical meanings. The present results accordingly support further study of automated language analysis as an assessment adjunct rather than its immediate clinical implementation: no classification accuracy, prospective prediction, or decision-analytic benefit was tested (Corcoran et al., 2018).

Future studies should use longitudinal or repeated-measures designs to determine whether changes in affective language accompany changes in symptom severity or treatment response, and independent validation samples are needed before any claim regarding clinical prediction can be considered. Increasing the amount of language per participant, through additional prompts or repeated assessments, may improve the reliability of low-frequency categories such as anger and sadness and would also allow a fairer test of the cognitive-process categories that were uninformative here. Turkish-language research would further benefit from combining LIWC with morphology-aware and context-sensitive methods: a morphological parser could quantify person marking more comprehensively than TotalI, while contextual embeddings could distinguish self-directed anger, interpersonal hostility, and negated emotional expressions (Kerz et al., 2023). Finally, the incremental value of language should be tested against stronger clinical baselines, including clinician-rated severity and functional impairment.

## 5 Conclusion

Affective language in structured sentence-completion responses was associated with dimensional symptom burden in clinically referred adolescents. General negative-emotion language showed the most consistent associations across depressive, emotional, behavioral, and total difficulties. When negative-emotion language was decomposed into non-overlapping components, anger and sadness each added information beyond the remaining negative-emotion words, and their contributions differed by outcome: the anger coefficient exceeded the sadness coefficient for conduct problems, whereas the two were comparable for depressive and emotional symptoms. Because each comparison was made within its own model, a direct cross-outcome test and replication remain necessary. Dictionary-detected first-person singular reference was unrelated to symptoms despite being sensitive to referential context, indicating that the English-language I-talk effect was not observed with the present morphology-informed operationalization. Whether the effect is absent in Turkish more generally, or whether it resides in forms of self-reference that a fixed dictionary cannot enumerate, remains an open question. The exploratory association between self-referential negativity and emotional symptoms further suggests that both emotional content and elicitation context may matter, although replication is required. Overall, automated analysis of brief Turkish sentence-completion responses appears feasible and informative at the group level, but its clinical value and cross-linguistic validity require longitudinal, morphology-aware, and independently validated research.

## Data Availability

De-identified derived data (LIWC category scores and clinical scale scores) are available from the corresponding authors on reasonable request. The raw sentence-completion texts cannot be shared, as they are free-text responses from minors that may contain identifying content and are covered by the terms of the ethics approval and participant consent.
