# Supplementary material for: Affective language and transdiagnostic symptom burden in clinically referred adolescents: a multicenter Turkish sentence-completion study
Source: Front Psychol. 2026 Sep 21;17:1958650. doi: 10.3389/fpsyg.2026.1958650 (PMC13635330; doi:10.3389/fpsyg.2026.1958650)
Supplement: Supplementary file 1 [file Table_1.docx]

# **Supplementary Material**

## **Supplementary Table S1. Content domains and thematic classification of the 24 sentence-completion items**

| **Item** | **Content domain** | **Theme used in secondary analyses** |
| --- | --- | --- |
| 1 | Early childhood | Whole-response analysis only |
| 2 | School | Whole-response analysis only |
| 3 | Growing up / future | Whole-response analysis only |
| 4 | Happiest occasion | Whole-response analysis only |
| 5 | Incomprehension | Whole-response analysis only |
| 6 | Boys | Whole-response analysis only |
| 7 | What saddens the participant | Negative-emotion elicitors |
| 8 | Mother | Family |
| 9 | Wishes (general) | Whole-response analysis only |
| 10 | Recurrent thoughts | Whole-response analysis only |
| 11 | Girls | Whole-response analysis only |
| 12 | Hypothetical agency | Whole-response analysis only |
| 13 | Being alone | Whole-response analysis only |
| 14 | Shame | Negative-emotion elicitors |
| 15 | Siblings | Family |
| 16 | Self (negative framing) | Self |
| 17 | Father | Family |
| 18 | Fear | Negative-emotion elicitors |
| 19 | Self in comparison with others | Self |
| 20 | Wishes regarding parents | Family |
| 21 | Loss of self-restraint | Whole-response analysis only |
| 22 | Disgust | Negative-emotion elicitors |
| 23 | Unforgettable event | Whole-response analysis only |
| 24 | Self-directed wish | Self |

*Note. The task was adapted from the short form of the Beier Sentence Completion Test (Form A). Because the instrument is a published adaptation, the verbatim Turkish item wording is not reproduced here; each item is identified by its content domain instead. Only participant-generated completions were included in the linguistic analyses, and the standardized stems were excluded. All 24 completions contributed to the whole-response analysis. Theme-based analyses used only the items classified as Family, Self, or Negative-emotion elicitors.*

## **Supplementary Table S2. Full coefficients for the primary hierarchical regression models (whole-response word count ≥ 25)**

**Panel A. CDI total (n = 416)**

| **Predictor** | **B** | **SE (OLS)** | **SE (HC3)** | **β** | **t (HC3)** | **p (HC3)** | **VIF** |
| --- | --- | --- | --- | --- | --- | --- | --- |
| Constant | 5.981 | 4.280 | 4.239 | — | 1.41 | 0.1583 | — |
| Age | 0.856 | 0.211 | 0.224 | 0.183 | 3.83 | 0.0001 | 1.11 |
| Sex | −3.152 | 0.908 | 0.941 | −0.166 | −3.35 | 0.0008 | 1.25 |
| Whole-response word count | −0.007 | 0.018 | 0.019 | −0.017 | −0.35 | 0.7235 | 1.19 |
| Koç site indicator | −0.252 | 0.960 | 0.953 | −0.012 | −0.26 | 0.7911 | 1.24 |
| Uludağ site indicator | −0.011 | 1.055 | 1.119 | 0.000 | −0.01 | 0.9923 | 1.21 |
| Other negative emotion | 0.540 | 0.171 | 0.172 | 0.137 | 3.13 | 0.0017 | 1.03 |
| Positive emotion | −0.312 | 0.092 | 0.093 | −0.155 | −3.36 | 0.0008 | 1.15 |
| Anger, square-root transformed | 1.942 | 0.441 | 0.428 | 0.193 | 4.54 | <.0001 | 1.06 |
| Sadness, square-root transformed | 2.329 | 0.488 | 0.500 | 0.216 | 4.66 | <.0001 | 1.12 |

Model statistics: R² = .259; adjusted R² = .243; ΔR² for Block 2 = .133, F(4, 406) = 18.20, p < .001. Anger versus sadness contrast: p = .721.

**Panel B. SDQ Emotional Symptoms (n = 421)**

| **Predictor** | **B** | **SE (OLS)** | **SE (HC3)** | **β** | **t (HC3)** | **p (HC3)** | **VIF** |
| --- | --- | --- | --- | --- | --- | --- | --- |
| Constant | 2.844 | 1.207 | 1.183 | — | 2.40 | 0.0162 | — |
| Age | 0.158 | 0.059 | 0.060 | 0.119 | 2.62 | 0.0087 | 1.11 |
| Sex | −1.365 | 0.257 | 0.266 | −0.254 | −5.14 | <.0001 | 1.27 |
| Whole-response word count | 0.005 | 0.005 | 0.005 | 0.046 | 0.98 | 0.3257 | 1.20 |
| Koç site indicator | −0.478 | 0.271 | 0.288 | −0.083 | −1.66 | 0.0966 | 1.23 |
| Uludağ site indicator | 0.424 | 0.297 | 0.291 | 0.067 | 1.46 | 0.1445 | 1.21 |
| Other negative emotion | 0.198 | 0.049 | 0.055 | 0.176 | 3.59 | 0.0003 | 1.03 |
| Positive emotion | −0.078 | 0.026 | 0.027 | −0.137 | −2.91 | 0.0037 | 1.16 |
| Anger, square-root transformed | 0.302 | 0.125 | 0.123 | 0.106 | 2.46 | 0.0140 | 1.05 |
| Sadness, square-root transformed | 0.470 | 0.138 | 0.141 | 0.154 | 3.33 | 0.0009 | 1.12 |

Model statistics: R² = .259; adjusted R² = .243; ΔR² for Block 2 = .089, F(4, 411) = 12.41, p < .001. Anger versus sadness contrast: p = .467.

**Panel C. SDQ Conduct Problems (n = 421)**

| **Predictor** | **B** | **SE (OLS)** | **SE (HC3)** | **β** | **t (HC3)** | **p (HC3)** | **VIF** |
| --- | --- | --- | --- | --- | --- | --- | --- |
| Constant | 1.848 | 0.915 | 0.870 | — | 2.12 | 0.0337 | — |
| Age | 0.035 | 0.045 | 0.045 | 0.039 | 0.79 | 0.4288 | 1.11 |
| Sex | −0.010 | 0.194 | 0.201 | −0.003 | −0.05 | 0.9583 | 1.27 |
| Whole-response word count | −0.004 | 0.004 | 0.004 | −0.055 | −1.06 | 0.2910 | 1.20 |
| Koç site indicator | 0.057 | 0.205 | 0.208 | 0.014 | 0.27 | 0.7844 | 1.23 |
| Uludağ site indicator | −0.037 | 0.225 | 0.227 | −0.009 | −0.16 | 0.8701 | 1.21 |
| Other negative emotion | 0.079 | 0.037 | 0.036 | 0.102 | 2.17 | 0.0299 | 1.03 |
| Positive emotion | −0.030 | 0.020 | 0.019 | −0.077 | −1.58 | 0.1142 | 1.16 |
| Anger, square-root transformed | 0.468 | 0.094 | 0.089 | 0.240 | 5.24 | <.0001 | 1.05 |
| Sadness, square-root transformed | 0.155 | 0.104 | 0.107 | 0.074 | 1.45 | 0.1467 | 1.12 |

Model statistics: R² = .087; adjusted R² = .067; ΔR² for Block 2 = .081, F(4, 411) = 9.16, p < .001. Anger versus sadness contrast: p = .011.

*Note.* B = unstandardized coefficient; β = standardized coefficient. Both ordinary least-squares and heteroscedasticity-consistent (HC3) standard errors are reported; all inference in the main text is based on HC3. Sex was coded 1 = female, 2 = male. Site indicators are dummy variables with Şişli Hamidiye Etfal Training and Research Hospital as the reference category. Other negative emotion denotes negative-emotion words remaining after anger and sadness words were removed. The anger versus sadness contrast is an HC3 Wald test of the difference between the two standardized coefficients within the same model, estimated on the standardized solution; the corresponding false-discovery-rate-adjusted q-values are reported in Table 3 of the main text. False-discovery-rate-adjusted q-values for the Block 2 coefficients are reported in Table 3 of the main text.

## **Supplementary Table S3. Regression sensitivity analyses without the minimum word-count criterion**

**Panel A. CDI total (n = 435)**

| **Predictor** | **B** | **SE (OLS)** | **SE (HC3)** | **β** | **t (HC3)** | **p (HC3)** | **VIF** |
| --- | --- | --- | --- | --- | --- | --- | --- |
| Age | 0.848 | 0.203 | 0.213 | 0.184 | 3.98 | 0.0001 | 1.10 |
| Sex | −3.021 | 0.872 | 0.897 | −0.161 | −3.37 | 0.0008 | 1.23 |
| Whole-response word count | 0.006 | 0.017 | 0.016 | 0.017 | 0.37 | 0.7120 | 1.21 |
| Koç site indicator | −0.231 | 0.944 | 0.936 | −0.011 | −0.25 | 0.8053 | 1.25 |
| Uludağ site indicator | −0.376 | 1.005 | 1.042 | −0.017 | −0.36 | 0.7178 | 1.23 |
| Other negative emotion | 0.408 | 0.148 | 0.142 | 0.119 | 2.87 | 0.0041 | 1.06 |
| Positive emotion | −0.301 | 0.086 | 0.089 | −0.158 | −3.37 | 0.0007 | 1.17 |
| Anger, square-root transformed | 1.998 | 0.420 | 0.405 | 0.206 | 4.94 | <.0001 | 1.06 |
| Sadness, square-root transformed | 2.055 | 0.454 | 0.467 | 0.201 | 4.40 | <.0001 | 1.11 |

R² = .252; adjusted R² = .236; ΔR² = .131, p < .001. Anger versus sadness contrast: p = .934.

**Panel B. SDQ Emotional Symptoms (n = 440)**

| **Predictor** | **B** | **SE (OLS)** | **SE (HC3)** | **β** | **t (HC3)** | **p (HC3)** | **VIF** |
| --- | --- | --- | --- | --- | --- | --- | --- |
| Age | 0.160 | 0.058 | 0.060 | 0.122 | 2.66 | 0.0079 | 1.10 |
| Sex | −1.265 | 0.250 | 0.268 | −0.236 | −4.73 | <.0001 | 1.24 |
| Whole-response word count | 0.010 | 0.005 | 0.005 | 0.096 | 2.03 | 0.0423 | 1.21 |
| Koç site indicator | −0.474 | 0.270 | 0.283 | −0.082 | −1.68 | 0.0936 | 1.24 |
| Uludağ site indicator | 0.221 | 0.287 | 0.286 | 0.036 | 0.77 | 0.4399 | 1.23 |
| Other negative emotion | 0.157 | 0.042 | 0.048 | 0.160 | 3.24 | 0.0012 | 1.06 |
| Positive emotion | −0.070 | 0.025 | 0.026 | −0.129 | −2.65 | 0.0081 | 1.18 |
| Anger, square-root transformed | 0.357 | 0.120 | 0.120 | 0.128 | 2.98 | 0.0028 | 1.06 |
| Sadness, square-root transformed | 0.404 | 0.130 | 0.136 | 0.138 | 2.97 | 0.0029 | 1.12 |

R² = .240; adjusted R² = .224; ΔR² = .085, p < .001. Anger versus sadness contrast: p = .874.

**Panel C. SDQ Conduct Problems (n = 440)**

| **Predictor** | **B** | **SE (OLS)** | **SE (HC3)** | **β** | **t (HC3)** | **p (HC3)** | **VIF** |
| --- | --- | --- | --- | --- | --- | --- | --- |
| Age | 0.040 | 0.044 | 0.043 | 0.044 | 0.92 | 0.3554 | 1.10 |
| Sex | 0.019 | 0.188 | 0.198 | 0.005 | 0.09 | 0.9249 | 1.24 |
| Whole-response word count | −0.002 | 0.004 | 0.004 | −0.034 | −0.66 | 0.5120 | 1.21 |
| Koç site indicator | 0.041 | 0.204 | 0.204 | 0.010 | 0.20 | 0.8419 | 1.24 |
| Uludağ site indicator | −0.060 | 0.216 | 0.218 | −0.014 | −0.28 | 0.7832 | 1.23 |
| Other negative emotion | 0.080 | 0.032 | 0.034 | 0.119 | 2.35 | 0.0187 | 1.06 |
| Positive emotion | −0.020 | 0.019 | 0.018 | −0.055 | −1.11 | 0.2679 | 1.18 |
| Anger, square-root transformed | 0.442 | 0.091 | 0.086 | 0.231 | 5.14 | <.0001 | 1.06 |
| Sadness, square-root transformed | 0.091 | 0.098 | 0.101 | 0.045 | 0.90 | 0.3675 | 1.12 |

R² = .080; adjusted R² = .061; ΔR² = .076, p < .001. Anger versus sadness contrast: p = .003.

*Note.* Models are identical to those in Supplementary Table S2 except that all participants with available data were retained, irrespective of whole-response word count. Constants are omitted for brevity.

## **Supplementary Table S4. Partial-correlation sensitivity analyses without the minimum word-count criterion**

| **LIWC category** | **CDI total** | **SDQ Total Difficulties** | **SDQ Emotional Symptoms** | **SDQ Conduct Problems** |
| --- | --- | --- | --- | --- |
| Negative emotion (total) | .333*** | .302*** | .275*** | .232*** |
| Positive emotion | −.232*** | −.208*** | −.192*** | −.096 |
| Anger, square-root transformed | .243*** | .231*** | .162** | .245*** |
| Sadness, square-root transformed | .207*** | .145** | .148** | .031 |
| First-person singular reference (TotalI) | −.020 | .034 | .005 | .032 |

*Note.* n = 430. Partial correlations controlled for age, sex, whole-response word count, and study site. The Benjamini–Hochberg correction was applied across this family of 20 tests. The positive emotion–Conduct Problems association did not survive correction (q = .063). **q < .01. ***q < .001.

## **Supplementary Table S5. Three-step hierarchical regressions testing the incremental contribution of anger and sadness language**

| **Outcome** | **n** | **R² Block 1** | **R² Block 2** | **R² Block 3** | **ΔR² Block 2 (F, p)** | **ΔR² Block 3 (F, p)** |
| --- | --- | --- | --- | --- | --- | --- |
| CDI total | 416 | .126 | .185 | .259 | .059 (F(2, 408) = 14.71, p < .001) | .074 (F(2, 406) = 20.30, p < .001) |
| SDQ Emotional Symptoms | 421 | .170 | .228 | .259 | .059 (F(2, 413) = 15.73, p < .001) | .031 (F(2, 411) = 8.52, p < .001) |
| SDQ Conduct Problems | 421 | .006 | .028 | .087 | .023 (F(2, 413) = 4.84, p = .008) | .059 (F(2, 411) = 13.20, p < .001) |

*Note.* Block 1: age, sex, whole-response word count, and two dummy-coded study-site variables. Block 2 adds other negative emotion and positive emotion (general affective tone). Block 3 adds square-root-transformed anger and sadness. Specific emotion words therefore added significant variance beyond general affective tone for all three outcomes. Analyses were restricted to participants with whole-response word count ≥ 25.

## **Supplementary Table S6. Descriptive statistics of the primary LIWC variables (whole-response sample, word count ≥ 25)**

| **LIWC variable** | **n** | **M** | **SD** | **Range** | **% zero** |
| --- | --- | --- | --- | --- | --- |
| Negative emotion (total) | 423 | 7.49 | 4.16 | 0.00–23.26 | 3.3 |
| Other negative emotion | 423 | 2.54 | 2.38 | 0.00–14.71 | 18.9 |
| Anger | 423 | 2.52 | 2.56 | 0.00–18.60 | 28.1 |
| Sadness | 423 | 2.43 | 2.19 | 0.00–11.11 | 25.8 |
| Positive emotion | 423 | 10.28 | 4.70 | 1.59–32.35 | 0.0 |
| First-person singular reference (TotalI) | 420 | 13.16 | 4.59 | 2.94–29.63 | 0.0 |

*Note.* Values are percentages of total words within each participant's concatenated whole-response text. Other negative emotion denotes negative-emotion words remaining after anger and sadness words were removed. TotalI aggregates overt first-person pronouns and first-person verb inflections; the overt-pronoun subcomponent alone averaged 0.38% of words and was zero in 78% of participants. Anger and sadness were square-root transformed for inferential analyses because of positive skew.

## **Supplementary Table S7. Partial correlations before and after adjustment for diagnostic domains**

| **LIWC category** | **Outcome** | **r, demographic covariates only** | **r, additionally adjusted for diagnostic domains** | **p** | **n** |
| --- | --- | --- | --- | --- | --- |
| Negative emotion (total) | CDI total | .346 | .297 | <.001 | 409 |
| Negative emotion (total) | SDQ Emotional Symptoms | .282 | .223 | <.001 | 414 |
| Negative emotion (total) | SDQ Conduct Problems | .248 | .249 | <.001 | 414 |
| Positive emotion | CDI total | −.220 | −.193 | <.001 | 409 |
| Positive emotion | SDQ Emotional Symptoms | −.191 | −.172 | .0004 | 414 |
| Positive emotion | SDQ Conduct Problems | −.117 | −.110 | .025 | 414 |
| Anger, square-root transformed | CDI total | .218 | .224 | <.001 | 409 |
| Anger, square-root transformed | SDQ Emotional Symptoms | .125 | .114 | .020 | 414 |
| Anger, square-root transformed | SDQ Conduct Problems | .242 | .257 | <.001 | 414 |
| Sadness, square-root transformed | CDI total | .228 | .185 | .0002 | 409 |
| Sadness, square-root transformed | SDQ Emotional Symptoms | .172 | .120 | .014 | 414 |
| Sadness, square-root transformed | SDQ Conduct Problems | .074 | .065 | .190 | 414 |

*Note.* All partial correlations controlled for age, sex, whole-response word count, and study site. The adjusted models additionally controlled for the four non-mutually-exclusive diagnostic-domain indicators (attention-deficit/hyperactivity disorder, other neurodevelopmental disorders, internalizing disorders, and other diagnoses). p-values are for the diagnosis-adjusted correlations. Analyses were restricted to participants with whole-response word count ≥ 25.

## **Supplementary Table S8. Site-stratified partial correlations for the principal associations**

| **Association** | **Koç University Hospital (n = 133–134)** | **Bursa Uludağ University (n = 96)** | **Şişli Hamidiye Etfal Training and Research Hospital (n = 186–192)** |
| --- | --- | --- | --- |
| Negative emotion – CDI total | .310 (p < .001) | .349 (p < .001) | .362 (p < .001) |
| Negative emotion – SDQ Emotional Symptoms | .125 (p = .150) | .325 (p = .001) | .360 (p < .001) |
| Negative emotion – SDQ Conduct Problems | .270 (p = .002) | .157 (p = .126) | .278 (p < .001) |
| Anger – SDQ Conduct Problems | .235 (p = .006) | .060 (p = .562) | .345 (p < .001) |

*Note.* Partial correlations controlled for age, sex, and whole-response word count within each site; the study-site covariate is not applicable in stratified models. Analyses were restricted to participants with whole-response word count ≥ 25. Sample sizes vary slightly across outcomes because of analysis-specific listwise deletion. The principal association (negative-emotion language with depressive symptoms) was of comparable magnitude at all three sites.

**Supplementary Table S9. Exploratory dictionary-wide screening of the remaining Turkish LIWC categories**

| **LIWC category** | **Coding** | **CDI total** | **SDQ Total Difficulties** | **SDQ Emotional Symptoms** | **SDQ Conduct Problems** |
| --- | --- | --- | --- | --- | --- |
|  |  | ***r (q)*** | ***r (q)*** | ***r (q)*** | ***r (q)*** |
| Cognitive mechanisms | Continuous | .026 (.881) | .109 (.503) | .093 (.729) | .088 (.778) |
| Insight | Continuous (sqrt) | −.120 (.503) | −.049 (.881) | −.033 (.881) | −.059 (.881) |
| Causation | Binary | .027 (.881) | −.018 (.885) | −.011 (.908) | −.039 (.881) |
| Certainty | Continuous (sqrt) | −.006 (.957) | −.045 (.881) | −.054 (.881) | .021 (.881) |
| Discrepancy | Continuous | .032 (.881) | −.011 (.908) | −.038 (.881) | .029 (.881) |
| Tentativeness | Continuous | .000 (.995) | .051 (.881) | .057 (.881) | .024 (.881) |
| Negations | Continuous | −.059 (.881) | −.044 (.881) | −.045 (.881) | .024 (.881) |
| Third-person reference | Continuous | −.024 (.881) | .006 (.957) | .031 (.881) | −.014 (.908) |
| Social words | Continuous | .019 (.885) | .045 (.881) | .055 (.881) | −.068 (.881) |
| Family | Continuous (sqrt) | −.005 (.957) | −.023 (.881) | −.020 (.881) | −.011 (.908) |
| Human | Continuous (sqrt) | .037 (.881) | −.014 (.908) | −.014 (.908) | −.042 (.881) |
| Achievement | Continuous (sqrt) | .074 (.881) | .020 (.881) | −.052 (.881) | .067 (.881) |
| Work | Continuous | .035 (.881) | .021 (.881) | −.033 (.881) | .049 (.881) |
| Desire | Continuous | .014 (.908) | .033 (.881) | .035 (.881) | .027 (.881) |
| Anxiety | Binary | .005 (.957) | .085 (.778) | .079 (.881) | .052 (.881) |
| Friends | Binary | .032 (.881) | .037 (.881) | −.003 (.963) | .003 (.963) |
| Health | Binary | .031 (.881) | .028 (.881) | .051 (.881) | −.030 (.881) |
| Death | Binary | −.070 (.881) | −.114 (.503) | −.101 (.576) | −.108 (.503) |
| Future focus | Binary | .023 (.881) | −.048 (.881) | .013 (.908) | −.075 (.881) |

*Note.* Values are partial correlations (r) with Benjamini–Hochberg false-discovery-rate-adjusted p-values (q) in parentheses. All analyses controlled for age, sex, whole-response word count, and study site (two dummy-coded variables), and were restricted to participants with a whole-response word count of at least 25 words. Analysis-specific listwise deletion was used; n ranged from 413 to 421. Categories with a high proportion of zero values were represented as binary presence indicators; the remaining categories were entered as percentages of total words, square-root transformed where indicated. These 76 tests were corrected as a single exploratory false-discovery-rate family, separate from the five confirmatory families defined in Section 2.4. No association survived correction (minimum q = .503), and all partial correlations were smaller than .12 in absolute value. Five tests reached nominal significance before correction (death–SDQ Total Difficulties, death–SDQ Conduct Problems, insight–CDI total, cognitive mechanisms–SDQ Total Difficulties, and death–SDQ Emotional Symptoms), which is within the number expected by chance across 76 tests. The affective and self-referential categories examined in the confirmatory analyses are reported in Table 2 of the main text and are not repeated here.

**Supplementary Table S10. Exploratory partial correlations between the primary linguistic variables and the SDQ Hyperactivity/Inattention and Peer Problems subscales**

| **LIWC category** | **SDQ Hyperactivity/Inattention** | **SDQ Peer Problems** |
| --- | --- | --- |
|  | ***r (q)*** | ***r (q)*** |
| Negative emotion (total) | .104 (.059) | .279 (<.001)*** |
| Positive emotion | −.103 (.059) | −.169 (.002)** |
| Anger, square-root transformed | .058 (.297) | .184 (.001)*** |
| Sadness, square-root transformed | .079 (.156) | .139 (.011)* |
| First-person singular reference (TotalI) | .031 (.522) | .039 (.469) |

*Note.* n = 417. Values are partial correlations (r) with Benjamini–Hochberg false-discovery-rate-adjusted p-values (q) in parentheses. All analyses controlled for age, sex, whole-response word count, and study site (two dummy-coded variables), and were restricted to participants with a whole-response word count of at least 25 words. These 10 tests were corrected as a single exploratory false-discovery-rate family, separate from the five confirmatory families defined in Section 2.4. The Hyperactivity/Inattention and Peer Problems subscales were not part of the pre-specified analyses and are reported here to document the full pattern across the SDQ. Peer Problems showed the same pattern of associations as the pre-specified outcomes, whereas no association with Hyperactivity/Inattention survived correction, despite 45.2% of the sample falling within the attention-deficit/hyperactivity disorder diagnostic domain. *q < .05. **q < .01. ***q < .001.
